# The Role of Endogenous Metal Nanoparticles in Biological Systems

**DOI:** 10.3390/biom11111574

**Published:** 2021-10-23

**Authors:** Vitaly Vodyanoy

**Affiliations:** Department of Anatomy, Physiology and Pharmacology, College of Veterinary Medicine, Auburn, AL 36849, USA; vodyavi@auburn.edu; Tel.: +1-334-844-5405

**Keywords:** proteons, blood, olfaction, cancer, prions

## Abstract

The blood and tissues of vertebrate animals and mammals contain small endogenous metal nanoparticles. These nanoparticles were observed to be composed of individual atoms of iron, copper, zinc, silver, gold, platinum, and other metals. Metal nanoparticles can bind proteins and produce proteinaceous particles called proteons. A small fraction of the entire pool of nanoparticles is usually linked with proteins to form proteons. These endogenous metal nanoparticles, along with engineered zinc and copper nanoparticles at subnanomolar levels, were shown to be lethal to cultured cancer cells. These nanoparticles appear to be elemental crystalline metal nanoparticles. It was discovered that zinc nanoparticles produce no odor response but increase the odor reaction if mixed with an odorant. Some other metal nanoparticles, including copper, silver, gold, and platinum nanoparticles, do not affect the responses to odorants. The sources of metal nanoparticles in animal blood and tissues may include dietary plants and gut microorganisms. The solid physiological and biochemical properties of metal nanoparticles reflect their importance in cell homeostasis and disease.

## 1. Introduction

Scientists of the 18th century observed and described particles found in human blood and other liquids [1,2]. Some of those forms could move and multiply. Recently, it was found that the blood and tissues of vertebrate animals and mammals contain endogenous metal nanoparticles comprising 30–400 atoms [3]. The metal nanoparticles were not oxidized or heterometallic but were composed of individual atoms of iron, copper, zinc, silver, gold, platinum, and other metals. Metal nanoparticles can bind proteins and produce proteinaceous particles of 0.05–5 µm in size called proteons. Some proteons exhibit a cell-like form with an external membrane-like structure, although their appearance is distinct from that of bacterial walls or the mammalian plasma membrane. Nanoparticles bind misfolded proteins and help to remove them from the blood. Endogenous metal nanoparticles obtained from the blood of humans, rabbits, or dogs and engineered zinc and copper nanoparticles at subnanomolar levels were shown to kill cultured cancer cells [3,4]. Naturally occurring zinc nanoparticles were found to exist in olfactory and nasal respiratory epithelia and cilia in animals [5]. Studies of these nanoparticles by transmission electron microscopy and the selected area electron diffraction method revealed the existence of metal elemental crystalline zinc nanoparticles 2–4 nm in diameter. The aim of this work is to review the physiological and biochemical properties of metal nanoparticles in living systems and to show their importance in both cell homeostasis and disease.

## 2. A Brief History

H. Charlton Bastian, who was an English physiologist and neurologist, observed particles produced within closed flasks that had previously been heated to 135 °C. Under the microscope, the particles moved, and their quantity increased. He believed that he had witnessed the spontaneous generation of living organisms out of non-living matter [1,2]. A few years earlier, Charles Darwin introduced small organic particles called gemmules aggregated in the gonads, contributing heritable information to the gametes [6,7]. In a letter to one of his closest friends, Joseph Dalton Hooker, Darwin wrote, “against all evidence, I cannot avoid suspecting that organic particles (my gemmules from the separate cells of the lower creatures) will keep alive [after boiling] and afterwards multiply under proper conditions. What an interesting problem it is” [8]. A French microbiologist Antoine Bechamp described small particles found in human and animal blood by using a high-resolution optical microscope. He called those small particles “microzymas.” He wrote, “microzymas, anatomical elements, are living beings of a special order without analogue” [9]. In 1925, Gunter Enderlein also described small particles found in the blood that he called protits. Protits are, according to Enderlein, small protein particles sized between 1 and 10 nm with inorganic nuclei. He stated that under certain conditions, protits could transform into other forms and sizes [10]. The discovery of small particles in blood was later reaffirmed by Wilhelm Reich [11], Royal Rife [12], and Gaston Naessens [13]. Yet, these seemingly identical forms described by different authors were not characterized to such a degree that allowed identifying them as the same or as distinct entities. By applying biochemistry and molecular biology methods, it was only recently possible to better describe and characterize nanoforms isolated from blood [14,15,16,17,18,19,20]. It now appears that nanoparticles found in the blood of vertebrate animals and humans, named nanobacteria [16], are not small bacteria but rather protein-associated mineralization [17,20].

## 3. Metal Nanoparticles in Blood and Tissues of Vertebrates and Mammals

**Figure 1:**
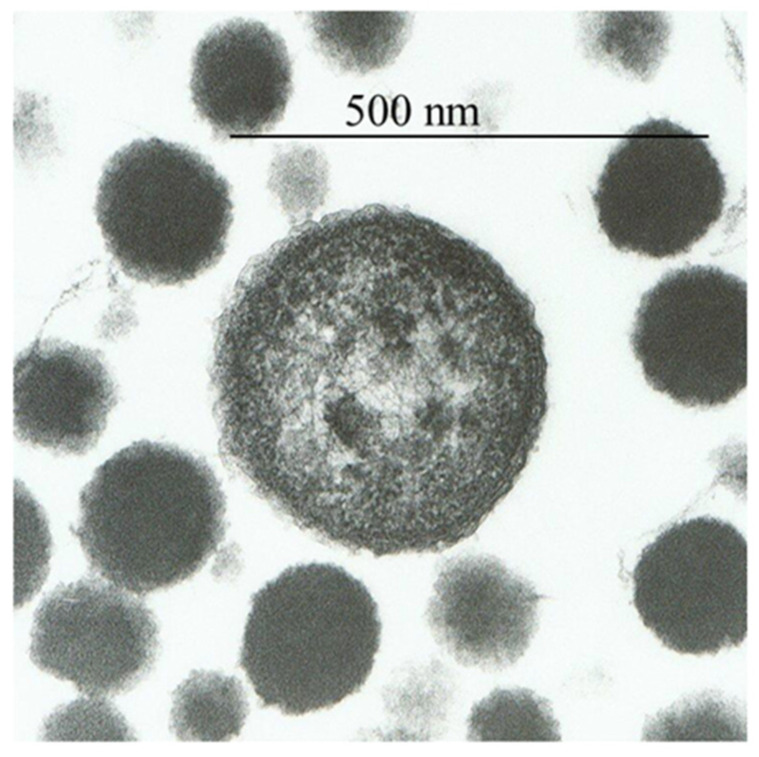
Electron micrographs of proteons from human blood.

The plasma of freshly drawn blood from humans, rabbits, dogs, or sharks was subjected to 120 °C at 140 kPa pressure for 2 h, revealing particles named proteons [3]. Healthy human blood contained ~3 × 10^8^ proteons/mL. Proteons 0.05–5 µm in size were visible by transmission and scanning electron microscopy as disks of 50 to 250 nm or coconut-shaped structures with one or more nuclei of the size of 1–5 µm (Figure 1). The larger proteons showed a cell-like form with a peripheral membrane-like structure, although their appearance was distinct from bacterial walls or the mammalian cell membrane. Instead, this external structure appeared as a fibrous shell with a thickness of 10–12 nm. Similar cell-like structures with the inner mineral core and the external simulated membrane were observed in calcified human arteries, cardiac valves [19], pathologic renal calcification [18], fetal bovine serum [14,20], and human atherosclerotic plaques [21]. Proteons from human blood are primarily composed of carbon, hydrogen, oxygen, nitrogen, sulfur, and minute levels of potassium, sodium, chlorine, zinc, and copper. Though proteons contain fragments of hemoglobin protein, they do not have nucleic acids [3]. Kajander and Ciftcioglu utilized the PCR technique to demonstrate the presence of DNA in nanobacteria from fetal bovine serum [14]. However, succeeding studies by Cisar and colleagues [20] reported that DNA found by Kajander and Ciftcioglu more likely belonged to *Phyllobacterium umyrsinacearum,* depicted as a typical contaminant in PCR studies [22].

**Figure 2:**
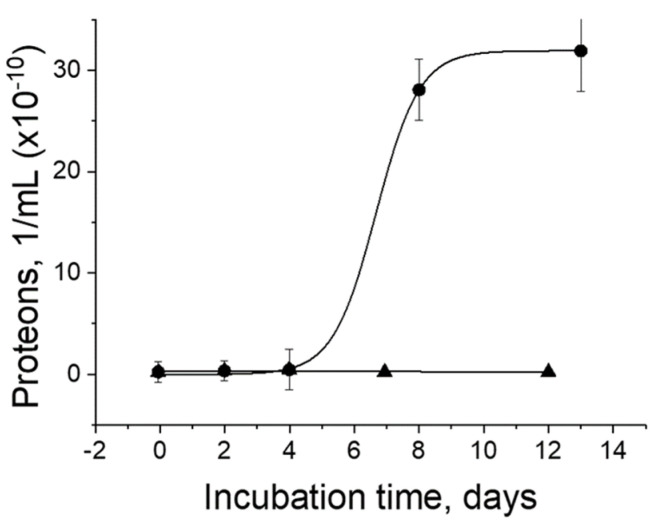
Proliferation of proteons. Proteons (●) were incubated in the tissue culture media (▲).

Proteons were incubated in culture medium proliferate, reaching saturation after 13 days [3]. The culture medium alone contained no proteons and served as a negative control (Figure 2). The half-time of proliferation is eight days.

The proliferation of proteons agrees well with the multiplication of the nanoforms obtained from fetal bovine serum in the culture medium, showing an S-shaped growth curve saturated in approximately 16 days [17].

Transmission electron microscopy and the selected area electron diffraction method revealed metal nanoparticles in blood plasma and inside proteons. Free metal nanoparticles of copper, zinc, and iron serve as nucleating centers for the nucleation and growth of proteons. Each milliliter of healthy human blood contains approximately 7 × 10^13^ crystalline non-oxidized metal nanoparticles, and only approximately 0.004% of the entire pool of nanoparticles is bound to form proteons. These nanoparticles are 1 to 2 nm in diameter and contain 30–400 atoms [3]. Similar nucleation and growth of nanoforms were described in fetal bovine serum [17].

The proliferation of proteons was observed under a high-resolution light microscope [23,24]. Early in the proliferation process, only a few motile proteons were observed, and the rest of the space around these proteons appeared clear and void. As time passed, even though there was no change in the number of proteons in the observation field, the average size of proteons slightly increased. The clarity of the solution was progressively reduced, and empty space was gradually filled with milky clouds that slowly turned into myriads of tiny particles that were faintly visible. These were not produced by division of original proteons—the numbers of those at this point were not changed—but instead originated from a large number of small particles invisible through light microscopy, becoming observable when their size reached the limit of detection of the microscope system. Gradually, tiny particles grew larger and completely filled the observation field. Following this, proteons started combining in groups, aggregating, and producing large proteons [25].

I was able to find an interesting description of particle formation in blood plasma, which was observed about 150 years ago. H. Charlton Bastian described new particles formed in blood plasma under continuous microscope observation: “It seems almost equally certain that they did not originate from particles which were recognizable by microscopic power employed, since the fluids were at first, to all appearance, perfectly homogeneous. Either, therefore, the minute particles which were seen at a later stage must have originated owing to some primitive formative process taking place in a really homogeneous organic solution, or else the fluid, seemingly homogeneous, in reality, contained the most minute particles (microscopically invisible), derived in some unknown way from the previously existing protoplasmic elements” [2]. While further describing particles of different sizes, Professor Bastian wrote: “The corpuscles also presented different aspects, the largest of them appeared to possess a cellular structure with slight evidence of a boundary wall, and numerous large protein granules within, more or less completely concealing a faint ovoid nuclear-looking body. This granular appearance seemed to become more and more marked as the corpuscles become larger, and the nucleus also becomes more and more distinct, though only appearing as a space free from granules. The corpuscles, which were about 10 microns in diameter and those that were of smaller size, presented none of these characters. There was no break whatever in the continuity of the series; all graduations in size could be and were measured from mere plastid particle of 250 nm in diameter up to fully developed corpuscle. But in those corpuscles which exceeded 10 microns, the protoplasm gradually become granular, and they then began to exhibit changes which appear characteristic of the age and approaching degeneration.” [2]. Professor Bastian also described particles similar to those observed in this study via electron microscopy (Figure 1) as particles that “had assumed a most distinctly cellular appearance—each cell containing one or perhaps two well-defined ovoid nuclei…” [2].

The protein scavenging properties of endogenous metal nanoparticles are critical for intravascular hemolysis, resulting in the release of erythrocyte contents like hemoglobin into the general blood circulation [26]. Hemolytic conditions with substantial intravascular hemolysis occur in many diseases, including sickle-cell disease and malaria [27]. Released hemoglobin is normally filtered in the renal glomeruli. A high hemoglobin load can cause kidney dysfunction [28]. Released hemoglobin is additionally captured by haptoglobin, which is then recognized by hemoglobin scavenger receptors and endocytosed by macrophages [29]. When haptoglobin is depleted during critically elevated hemolysis [27], the released hemoglobin is collected by endogenous metal nanoparticles. The scavenging capabilities of metal nanoparticles are very high. On average, a 160-nm diameter proteon can bind approximately 10,000 protein molecules that are the size of hemoglobin [3].

The process of misfolded protein scavenging may provide an understanding of the mechanisms of some blood conditions related to intravascular hemolysis, which causes hemoglobin aggregation [30,31,32]. Certainly, metals have vital functions in conformation-based disorders such as prion disease, Parkinson’s disease, Alzheimer’s disease, and familial amyotrophic lateral sclerosis [33].

## 4. Metal Nanoparticles and Prions

**Figure 3:**
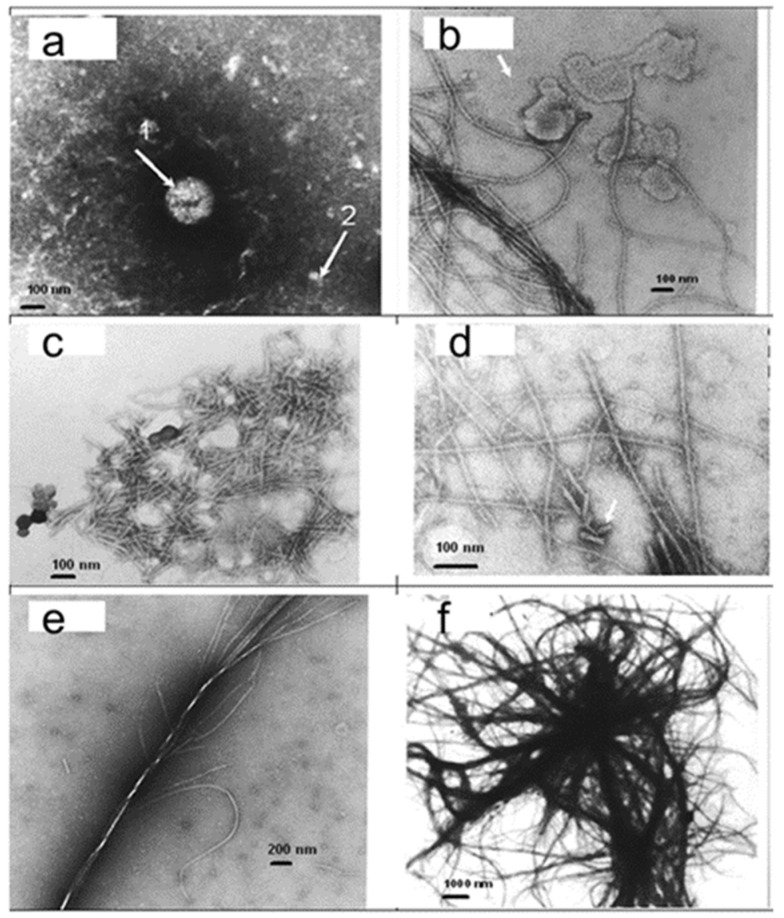
Effect of metal nanoparticles on crystallization of prion protein. Transmission electron microscopy. (**a**) Sample without metal nanoparticles. Arrow 1: A small number of relatively large round ball-type particles of ~300 nm. Arrow 2: A limited number of ~30 nm particles. (**b**) A large number of small particles (arrow) of 10–15 nm and filamentous structures were observed in the presence of metal nanoparticles. (**c**) Thin short rodlets (40–50 nm long). (**d**) Rodlets interconnected into longer thin filaments. (**e**) Thin filaments are interwoven into the thicker fibril. (**f**) Large fibrils create a system of fibrils and plaques.

Prions are misfolded proteins capable of transmitting their misfolded shape onto normal variants of the same protein [34]. The infectious prion PrP^Sc^ converts surrounding normal prion proteins (PrP^C^) by stimulating them to assume this abnormal conformation. PrP^Sc^ clusters accumulate within the cell and exit the cell into neighboring tissues. As aggregate-containing cells die, they form the ‘spongiform’ manifestation of transmissible spongiform encephalopathies [35]. Trace minerals, including manganese and zinc, have been shown to interact with PrPC and have been found in abnormal concentrations in prion diseases [36,37,38,39]. To examine the effect of metal nanoparticles on the crystallization of prion protein, recombinant human PrP(23–230) (Alicon, Schlieren, Switzerland) was dissolved in purified water at a concentration of 1 mg/mL at 25 °C and heated at 120 °C and 20 psi for 20 min. Then, 3 µL of metal nanoparticle suspension from shark blood at a concentration of 5 × 10^13^ 1/mL was added to 7 µL of PrP solution, and the sample was subjected to the same temperature and pressure treatment (Figure 3).

Transmission electron microscopy showed a few types of structures in PrP samples. In samples without metal nanoparticles, a small number of particles were observed. In the presence of metal nanoparticles, a dramatic change in the protein structure was observed. The structure changes from small particles to small rodlets, filaments, and thick fibrils, and finally, large fibrils create a complicated system of fibrils and plaques (observed in my laboratory, Figure 3). It took 160 h to grow fibrillary structures from recombinant PrP (89–231) at the University of California [40], while it took us only 15 min to produce all structures shown in Figure 3. I suggest that the difference in the rate of growth is explained by the presence of metal nanoparticles, so that those prion particles are formed by the nucleation and growth of proteins around nucleating centers [3].

## 5. Metal Nanoparticles Are Lethal to Cancer Cells

**Figure 4:**
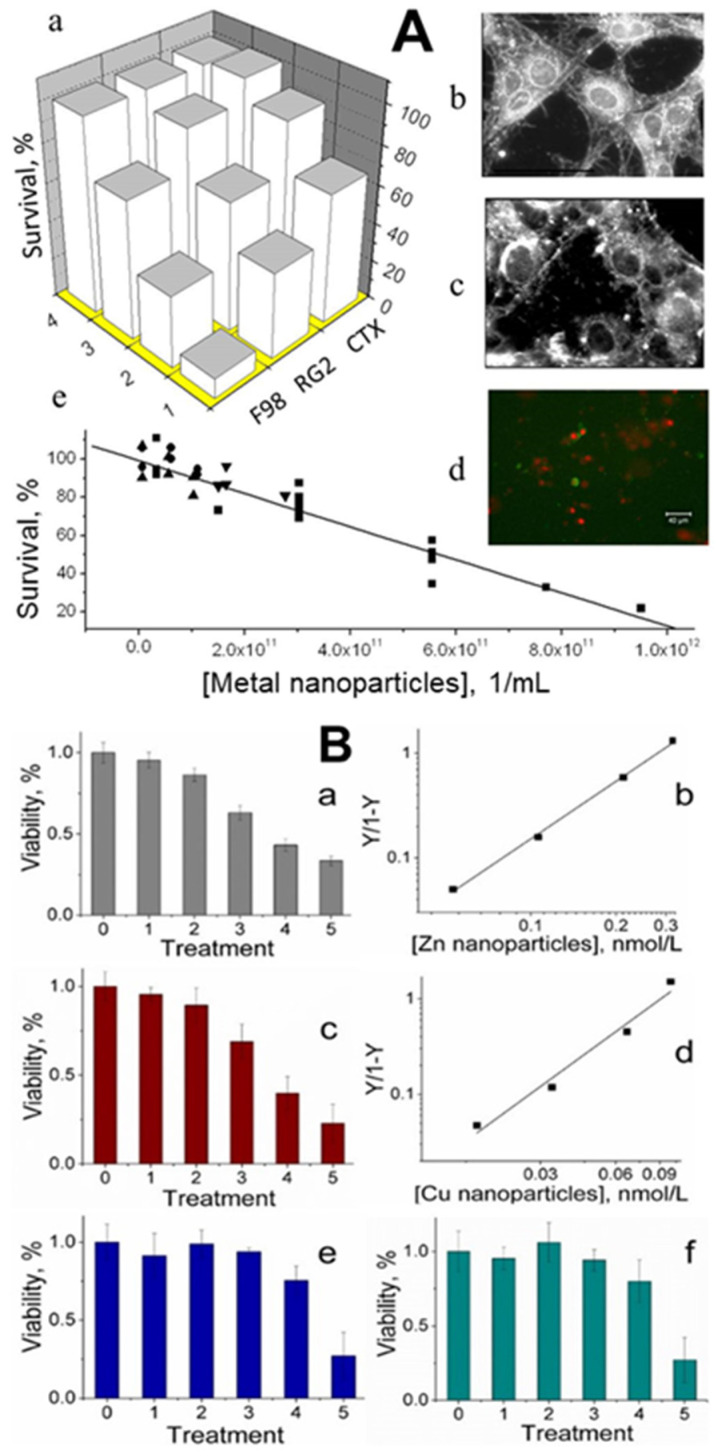
Panel (**A**). Effect of PNCs on cell viability. (**a**)—Viability of cultured cells after a 20-h exposure to different concentrations of metal nanoparticles (obtained from shark blood). Row 1: 1.7 × 10^11^ metal nanoparticles/mL; row 2: 9.1 × 10^10^; row 3: 9.9 × 10^9^; row 4: no metal nanoparticles. F98 and RG2 represent rat brain glioma cells, and CTX represents rat astrocytes. (**b**)—Darkfield microscope images of rat glioma cells. (**c**)—Cells after exposure to 7.7 × 10^11^ metal nanoparticles/mL. (**d**)—Fluorescence photomicrograph (400×) of RG2 glioma cells exposed to 7.7 × 10^11^ PNCs/mL for 30 min and stained with Annexin V and propidium iodide. (**e**)—Viability of cultured RG2 after 20 h of exposure to different concentrations of Pmetal nanoparticles obtained from the blood of blue sharks (Prionace; ■), dogs (●), humans (▲), or New Zealand white rabbits (Harland Sprague-Dawley) (▼). R^2^ = 0.95; *p* < 0.0001. Scale bars: 40 µm. Panel (**B**). Viability and mortality of glioma cancer cells treated with metal nanoparticles (error bars are standard deviations). (**a**)—Viability of glioma cells under six experimental conditions: 0, no treatment; 1, 2, 3, and 4, zinc nanoparticles (all expressed in nmol/L) 0.053, 0.106, 0.212, and 0.318, respectively; 5, 1 μmol/L of staurosporine. (**b**)—Hill representation of glioma cell mortality caused by zinc nanoparticles. (**c**)—Viability of glioma cells under six experimental conditions: 0, no treatment; 1, 2, 3, and 4, copper nanoparticles (all expressed in nmol/L) 0.017, 0.033, 0.066, and 0.1, respectively; 5, 1 μmol/L of staurosporine. (**d**)—Hill representation of glioma cell mortality caused by copper nanoparticles. (**e**)—Viability of astrocytes under six experimental conditions: 0, no treatment; 1, 2, 3, and 4, zinc nanoparticles (all expressed in nmol/L) 0.053, 0.106, 0.212, and 0.318, respectively; 5, 1 μmol/L of staurosporine. (**f**)—Viability of astrocytes under six experimental conditions: 0, no treatment; 1, 2, 3, and 4, copper nanoparticles (all expressed in nmol/L) 0.017, 0.033, 0.066, and 0.1, respectively; 5, 1 μmol/L of staurosporine. (Adopted from [4]).

Cancer is a disease that should not affect us if our defense systems are intact. All healthy animals and humans have small metal nanoparticles in their blood that may be dominant components of the body’s defense mechanisms against cancer. Small concentrations of metal nanoparticles of size 1–2 nm isolated from animal blood were observed to be toxic to cultured cancer cells. [3,4]. After incubation with these nanoparticles, the viability of two rat glioma cell lines (F98 and RG2) decreased by 90% and 75%, respectively, while that of normal rat astrocytes (CTX) was reduced by only 25%. Total suppression of growth in-vitro required ≈ 1 × 10^12^ metal nanoparticles/mL (i.e., a few nmol/L), a concentration smaller than what is typically found in a healthy animal [3] (Figure 4A). Engineered zinc and copper metal nanoparticles of size 1 nm–2 nm were lethal to cultured RG2 glioma cancer cells. Cell death was confirmed by a colorimetric assay to assess cell metabolic activity, showing that the relative viability of RG2 glioma cells was reduced in a dose-dependent manner at subnanomolar concentrations of the nanoparticles. Noncancerous astrocytes were not affected under the same conditions [4] (Figure 4B). Synthetic copper nanoparticles have been found to be toxic to cultured cancer cells, including U937 (human histiocytic lymphoma) and HeLa cells (human cervical cancer origin), at concentrations of 1–500 μmol/L, [41,42,43,44], which are a few orders of magnitude higher than those of engineered zinc and copper nanoparticles [4].

## 6. Nanoparticles in the Initial Events of Olfaction

**Figure 5:**
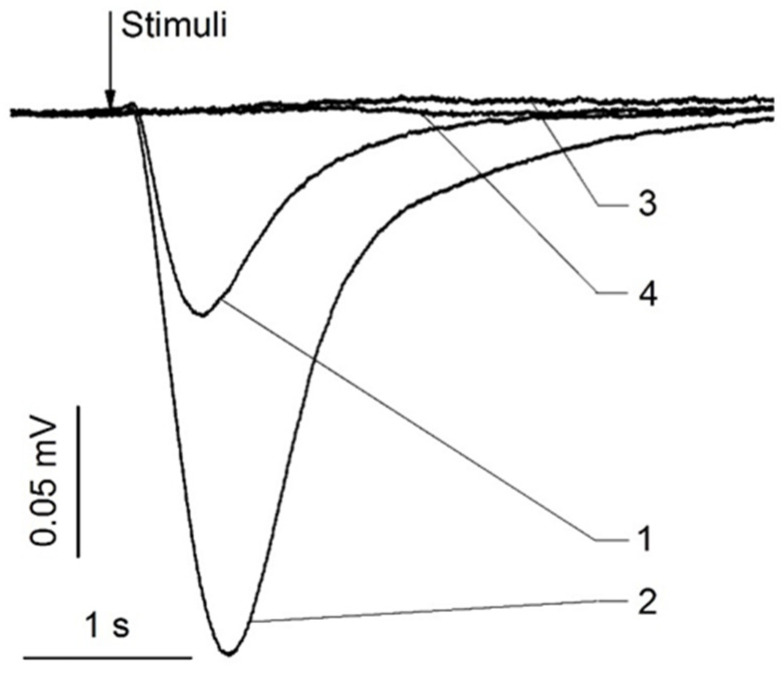
Representative electroolfactogram recordings from rat olfactory and respiratory epithelia. The stimuli were 0.25 s pulses of odorant with or without nanoparticles. (1) olfactory epithelium, odorant mixture, (2) olfactory epithelium, odorant mixture +1.2 nm zinc nanoparticles, (3) respiratory epithelium, odorant mixture, (4) respiratory epithelium, odorant mixture +1.2 nm zinc nanoparticles. The demonstrative set of traces was obtained from 200 EOG traces. Adopted from [45].

Animals have endogenous zinc nanoparticles in their olfactory and nasal respiratory epithelia and cilia [5]. The electrical responses of olfactory sensory receptors were dramatically boosted when these nanoparticles were added to a mixture of odorants, together with ethyl butyrate, eugenol, and carvone [5] (Figure 5).

Transmission electron microscopy and selected area electron diffraction analysis of these nanoparticles revealed metal elemental crystalline zinc nanoparticles with diameters of 2–4 nm. There is no oxidized zinc in these particles. The amplification caused by manufactured zinc nanoparticles is equivalent to the augmentation of odorant reactions induced by endogenous zinc nanoparticles [45,46,47]. Zinc nanoparticles have no odor when used alone, but when combined with an odorant, they enhance the odor response. These effects are reversible and dose-dependent. Copper, silver, gold, and platinum nanoparticles have none of the impacts that zinc nanoparticles have. Blood and other tissues were also discovered to contain endogenous zinc nanoparticles. Zinc, copper, and iron metal nanoparticles were detected after ultrafiltration of human and animal blood [3]. The mixture of these metal nanoparticles was also capable of enhancing olfactory responses to odorants [46].

The olfactory response was observed to be reduced when zinc nanoparticles were replaced with Zn^2+^ ions at the same concentrations. It was found that zinc nanoparticles function at the olfactory receptor level and are involved in the initial events of olfaction based on tests with cyclic adenosine monophosphate inhibitors [48]. The stoichiometry of metal nanoparticles and receptors, as well as the model of their function, were discovered by kinetic investigations of olfactory receptor/odorant/metal interactions [49]. The stoichiometry of the olfactory model requires that one metal nanoparticle binds two receptor molecules to form a dimer. Canine functional magnetic resonance imaging (fMRI) results show that the addition of zinc nanoparticles results in a considerable increase in brain activity in response to odorants, indicating that olfactory enhancement is recognized at the level of perception [50,51].

The fraction of olfactory receptors in cilia has been shown to be in the dimeric state, while the rest of the receptors remain in the monomeric state [52]. Endogenous zinc nanoparticles suspended in the cytoplasm of cilia are thought to be in equilibrium with nanoparticles attached to receptor dimers in the ciliary membrane [53]. Receptor dimers are essential because only dimeric receptors are active and take part in olfaction, whereas monomeric receptors are passive [53]. When the zinc level in the ciliary membrane or cytoplasm rises, unbound receptor monomers form new dimers [49]. Zinc nanoparticles are administered in combination with an odorant to increase the olfactory signal for a simple reason. These endogenous zinc nanoparticles produce a specific number of operational receptor dimers activated by the odorant and participate in olfactory signal initiation. The remaining monomeric receptors are inactive and do not contribute to the olfactory response elicited by odorants. When natural or manufactured zinc nanoparticles are delivered to the olfactory epithelium with the same odorant, new receptor dimers are generated from monomeric receptors. The addition of new receptor dimers will thereafter amplify the odorant-evoked olfactory response. An increase in zinc concentration aids the conversion of monomeric receptors into dimers until the monomers are depleted and the odorant-evoked olfactory response is saturated [5].

Suspensions of 1.2 nm fresh and oxidized zinc nanoparticles, as well as ZnO particles of various sizes, contained Zn^2+^ ions at different concentrations. We demonstrate that after oxidation, the changed physical state of zinc rather than the changed valence state is a result of the ZnO nanoparticles’ failure to enhance olfactory odorant responses [45]. It is worth noting that zinc oxide nanoparticles exhibit antimicrobial and antifungal activity and also show selective toxicity toward normal and cancerous cells [54].

The increase in the zinc nanoparticles in the odorant response was replicated at Zhejiang University [55]. An electrical signal generated by an odorant was significantly amplified when the manufactured zinc nanoparticles were introduced to an odorant as a rodent olfactory epithelium was placed on a microelectrode array.

The enhancement of the olfactory response by zinc nanoparticles is important in consideration of Luca Turin’s vibrational theory, which suggests inelastic electron tunneling spectroscopy (IETS) for discriminating odors [56]. The odorant triggers inelastic tunneling of an electron between donor and acceptor inside the receptor in the IETS process. Turin believes that the olfactory signal cascade is activated by electron tunneling. This action necessitates the presence of an electron donor, and zinc nanoparticles are a likely candidate [45,49].

The functional relevance of endogenous zinc nanoparticles in the early stages of olfaction [5] has been demonstrated in young and adult mouse olfactory epithelium cultures [57] in the dissected olfactory epithelium of rodents [5,45,46,47] and in live, conscious dogs [50,51].

## 7. Stability of Small Zinc Nanoparticles

The endogenous metal nanoparticles obtained from the blood and tissue of animals show high stability. They were capable of enhancing the olfactory response to odorant after storage at 5 °C for two years. We demonstrated that zinc nanoparticles, in the mixture of endogenous metals, are responsible for olfactory enhancement [5,46]. We studied the participation of zinc particles in olfaction. Zinc nanoparticles of ~1.2 nm in size were prepared by a high-voltage electrical discharge method [58]. Atomic force microscopy, transmission electron microscopy, and X-ray photoelectron spectroscopy, together with physiological animal experiments, were carried out [45,47]. Zinc nanoparticles were round and crystalline with a hexagonal close-packed lattice. The estimated 12 atoms of the core and the total 59 atoms of the 1.2 nm zinc nanoparticle are in close agreement with the “magic number” full-shell nanoparticles with 13 and 55 atoms. Each metal atom has the maximum number of nearest neighbors, which imparts some degree of extra stability to full-shell clusters [59,60]. After being kept at 5 °C in water for 24 h, freshly prepared zinc nanoparticles consisted of 97% non-oxidized atoms. After 300 days of storage in the same conditions, zinc nanoparticles lost only 1% of their atoms to oxidation and thus consisted of 96% non-oxidized zinc. Even with 48 h of storage at 50 °C, zinc nanoparticles contained 93% non-oxidized atoms [47]. Non-oxidized zinc nanoparticles maintained olfactory enhancement following 60 days of storage. After oxidation, zinc nanoparticles lose their ability to enhance responses to odorants [45]. A thin polyethylene glycol coating of zinc nanoparticles prolonged olfactory enhancement for over 300 days [47]. We suggest that the thin organic layer around the particles [5] can explain the long-term stability of the endogenous zinc nanoparticles.

## 8. Origin of Metal Nanoparticles in Live Systems

The presence of relatively high amounts of endogenous metal nanoparticles in animal blood and tissues raises questions about their origin. Plants, invertebrates, and microbes all have the ability to naturally reduce metal ions to neutral atoms and assemble them into metal nanoparticles [61]. It is unclear whether or not vertebrate animals possess this capability. Metal ions can be reduced to elemental metals by microbes in the gut microbiome. The reaction Zn^2+^ + 2e^−^ = Zn^0^, which is achieved via electron transfer [49,61], is used to reduce zinc ions into metal zinc. Electron transfer occurs in the intestine of mammals such as humans, mice, rats, and guinea pigs [62,63]. As a result, gut microbes producing zinc nanoparticles from zinc ions are a plausible idea. Recently, it has become more widespread in practice to create metal nanoparticles by microorganism synthesis. It was demonstrated that bacteria, fungi, and viruses could be utilized for the synthesis of metal nanoparticles [54,64]. The fact that zinc absorption from the diet is regulated by gut microorganisms [65] supports the credibility of this idea. Dietary plants are the critical source of zinc since they absorb it from the soil. Zinc is taken up by roots from the soil predominantly in the form of Zn^2+^ ions, which then translocate through the xylem to the above-ground parts of plants. Metal nanoparticles are formed when a portion of the zinc ions in a plant is reduced [66], leading the plant to provide zinc ions and zinc nanoparticles.

## 9. Optimal Size of Zinc Nanoparticles to Enhance Olfaction

Endogenous zinc nanoparticles in olfactory epithelia were found to have a diameter of 2–4 nm [5]. We can pause to consider the following question: what is the optimal size of zinc nanoparticles for enhancing the olfactory response? Large zinc nanoparticles have been demonstrated to have no effect on odorant responses [45]. We propose that particles should be small enough to have quantum properties. It has been predicted that nanoparticles with diameters of 1–10 nm would exhibit quantum-mechanical properties [67]. The physical properties that arise are not those of a bulk metal or a molecular compound. The particle size, interparticle distance, and shape of the nanoparticles, on the other hand, have a large impact on these properties [54,68]. If the de Broglie wavelength of the valence electrons is of the same order as the particle size, the quantum size effect is seen. As a result, the particles behave as zero-dimensional quantum dots (or quantum boxes) in electronic terms, which is very significant for quantum-mechanical principles. In contrast to bulk metals, nanoparticles have a gap between the valence and conduction bands.

Single-electron tunnel transitions occur between a donor and acceptor if the electrostatic energy, E_el_ = e^2^/2C, is larger than the thermal energy, E_T_ = kT, where C is the electric capacitance, e is the elementary charge, k is the Boltzmann coefficient, and T is the absolute temperature [69]. The capacitance of a 2 nm metal particle is C = 4πεε_o_r, where ε is the relative dielectric constant of the receptor protein [70], ε_o_ is the vacuum dielectric constant 8.85 × 10^−12^, and r = 1 nm. C = 4π × 9 × 8.85 × 10^−12^ × 10^−9^ = 1.0 × 10^−18^ F. E_el_ = (1.6 × 10^−19^)2/(2 × 1.0 × 10^−18^) = 1.15 × 10^−19^ CV = 79.6 meV, which is considerably higher than the thermal energy at 25 °C, E_T_ = 1.38 × 10^−23^ × 298 = 4.11 × 10^−21^ J = 25.6 meV. As a result, metal nanoparticles with a diameter less than 6 nm satisfy the single-electron transfer criterion [45]. At an ambient temperature, single-electron tunnel transition was observed with a 1 nm quantum dot [71]. Given this, the notion that small zinc nanoparticles act as electron donors (in Turin’s model [56]) appears to be highly plausible. In the physics of small metal particles studied by surface plasmon resonance, in the chemistry of supramolecular structures and molecular recognition, and in the biology of DNA-metal nanoparticle assemblies and sensors (reviewed in [69]), discontinuous size effects were experimentally observed.

## 10. Future, Concluding Remarks

Endogenous metal nanoparticles, as well as bound and free metal ions, are essential components of living organisms’ mineral constituents. Metals in living systems are thought to exist in two states: one form is tightly linked to metalloproteins and zinc finger proteins, whereas the other is a more labile ionic form that can be depleted via metal deprivation. Plants, microbes, and mammalian blood and tissues have all been found to have the third state of metal. Elemental, crystalline, non-oxidized metal nanoparticles with diameters of 2–4 nm have been observed to be functionally important in olfaction’s initial events. The presence of metal nanoparticles in the living system is significant, implying that metal nanoparticles are present in many more cells and tissues. The capacity of metal nanoparticles to scavenge misfolded proteins in the blood and destroy cancer cells suggests that these particles play an important role in maintaining the balance of living systems. Nanoparticles are simple objects of study as they may be engineered or harvested from live tissue, to study their interactions with living systems and observe endogenous metal particles in various species. This presents an opportunity to evaluate the transition from animal to human models and to study oncogenesis mechanisms of cell activation and inhibition, along with other cell interactions and communication processes. Animal and human studies of metal nanoparticles could lead to new approaches in oncology, as well as in other fields of research and medicine where metal nanoparticles can be used for therapeutic purposes.
